# Pharmacological Modulators of Endoplasmic Reticulum Stress in Metabolic Diseases

**DOI:** 10.3390/ijms17020192

**Published:** 2016-02-01

**Authors:** Tae Woo Jung, Kyung Mook Choi

**Affiliations:** Division of Endocrinology and Metabolism, Department of Internal Medicine, College of Medicine, Korea University, Seoul 152-703, Korea; ohayo2030@hanmail.net

**Keywords:** endoplasmic reticulum stress, unfolded protein response, AMPK-activated protein kinase, glucagon-like peptide-1, peroxisome proliferator-activated receptors, angiotensin II type 1 receptor blockers

## Abstract

The endoplasmic reticulum (ER) is the principal organelle responsible for correct protein folding, a step in protein synthesis that is critical for the functional conformation of proteins. ER stress is a primary feature of secretory cells and is involved in the pathogenesis of numerous human diseases, such as certain neurodegenerative and cardiometabolic disorders. The unfolded protein response (UPR) is a defense mechanism to attenuate ER stress and maintain the homeostasis of the organism. Two major degradation systems, including the proteasome and autophagy, are involved in this defense system. If ER stress overwhelms the capacity of the cell’s defense mechanisms, apoptotic death may result. This review is focused on the various pharmacological modulators that can protect cells from damage induced by ER stress. The possible mechanisms for cytoprotection are also discussed.

## 1. Introduction

The endoplasmic reticulum (ER) is observed in all eukaryotes and it is the site of protein folding, assembly of multi-subunit proteins, biosynthesis of lipids and sterols, and calcium storage. One major cellular stress is an augmented load of proteins targeted for secretion because the presence of too many unfolded proteins leads to an imbalance between the load of proteins entering the secretory pathway and the capacity of the ER to fold and process them, thereby leading to ER stress. To adapt to ER stress, a group of signal transduction proteins, termed the unfolded protein response (UPR), is activated. The UPR is mainly regulated by transmembrane ER-resident proteins such as inositol-requiring enzyme 1 (IRE1), PKR-like endoplasmic reticulum kinase (PERK), and activating transcription factor (ATF) 6. However, prolonged UPR leads to cell death via multiple apoptotic signaling cascades such as the CCAAT/enhancer-binding protein homologous protein (CHOP)-mediated pathway, IRE-1/tumor necrosis factor receptor-associated factor 2 (TRAF2)-mediated pathway, and Ca^2+^-associated signaling [1]. Although pharmacological modulators of ER stress usually protect cellular damage, literature on modulators is not always effective in establishing the link between ER-stress modulation and cellular damage. Furthermore, Varadarajan *et al.* demonstrated a new, evolutionarily conserved cellular stress response associated with reorganization of ER membrane that causes impairment of ER transport and function independently of the UPR [2].

Accumulated evidence demonstrates that ER stress is involved in the pathogenesis of protein misfolding disorders including neurodegenerative diseases (such as Parkinson’s and Alzheimer’s disease) and metabolic diseases (diabetes, cardiovascular disease, and non-alcoholic fatty liver). Therefore, the identification of pharmacological modulators is crucial for cytoprotection against cellular damage from ER stress. Based on previous reports, we have reviewed the protective effects of various drugs against cell damage caused by ER stress.

## 2. Cellular Aspects of ER-Stress and Metabolic Diseases

### 2.1. Diabetes Mellitus

Growing evidences support a critical role for activation of β-cell ER stress pathways in pathophysiology of diabetes [3]. Animal models of obesity and diabetes showed increased levels of ER stress, leading to insulin resistance and inflammatory responses [4]. Obesity has been reported to induce ER stress, which leads to the impairment of insulin signaling through hyperactivation of c-Jun N-terminal kinase (JNK)-mediated pathways [5]. Tersey *et al.* demonstrated that increased parameters of ER stress precede the onset of type 1 diabetes in isolated islets from prediabetic nonobese diabetic (NOD) mice [6]. Islet cells from 13 patients with type 1 diabetes revealed a partial ER stress response, including increased levels of CHOP [7]. Furthermore, PERK signaling is required to maintain endocrine function in pancreatic β-cells. Increased cell death and progressive diabetes mellitus with exocrine pancreatic insufficiency was observed in PERK knockout mice [8]. Similarly, conditional deletion of X-box binding protein 1 (XBP1) in pancreatic β-cells induced hyperglycemia and glucose intolerance resulting from reduced insulin secretion [9]. ER overload in β-cells induced ER stress which results in apoptosis via CHOP activation [10]. Targeted disruption of CHOP attenuated β-cell loss and delayed diabetes in the Akita mice, suggesting the pivotal role of the UPR in β-cell survival [10]. Thameem *et al.* reported that ATF6α polymorphisms are associated with type 2 diabetes in Pima Indians [11]. Elevations in the proinsulin/insulin ratio may be indicative of ER dysfunction in pancreatic β-cells, reflecting alterations in protein-folding and processing. Elevations in serum proinsulin/insulin ratio have been shown in patients with type 2 diabetes and those with new onset type 1 diabetes, while improvement in this ratio was reported following treatment with pioglitazone and IL-1β receptor antagonist therapy [3]. Glyburide treatment did not show further deleterious effects on ER stress or apoptosis of INS-1 cells in a glucotoxic condition [12].

### 2.2. Cardiovascular Diseases (CVD)

ER stress and UPR play major roles in the development and progression of CVD, including atherosclerosis, ischemic heart disease, and heart failure [13]. In ischemic-reperfusion injury, hypoxia and hypoglycemia caused by reduction of blood flow rapidly induce ER stress. Misfolded ER proteins are also caused by oxidative stress and alterations in the redox status of the ER in reperfusion of the affected tissues, when blood flow is recovered. Previous studies reported the protective roles of XBP-1 and ATF6 in ischemic/reperfusion injury, whereas activation of PERK/ATF4/CHOP pathway triggered apoptosis [14]. In apolipoprotein E-deficient mice, UPR markers were markedly increased in early intimal macrophages and in macrophage foam cells from advanced atherosclerotic lesions [15]. Saturated fatty acids, oxidized phospholipids, and oxidized low density lipoprotein (LDL) caused CD36-Toll-like receptor 2 (TLR2)-dependent apoptosis in ER-stressed macrophages, a key process in plaque necrosis [16].

### 2.3. Non-Alcoholic Fatty Liver Disease (NAFLD)

ER stress-mediated signal pathways have been shown to be associated with lipotoxicity, insulin resistance, inflammation, oxidative stress, and hepatic apoptosis, which are common properties of obesity and non-alcoholic fatty liver disease [17]. Elevated ER stress has been detected in liver of genetic and diet-induced non-alcoholic steatohepatitis (NASH) [18]. Furthermore, a variable degree of UPR activation was also documented in the liver of NAFLD or NASH patients [19]. Hepatic steatosis and lipogenesis are regulated by the PERK-eIF2α-ATF4 pathway [20]. Attenuated eIF2α in transgenic mouse liver was strongly correlated with suppression of adipogenesis-mediated regulators including peroxisome proliferate activated receptor-γ (PPAR-γ) and its upstream regulators, CCAAT/enhancer-binding proteins (C/EBP)-α and C/EBP-β [21]. ATF4-knockout mice showed protection from hypertriglyceridemia, diet-induced obesity, and hepatic steatosis [20]. In addition, the IRE1α-XBP1-mediated pathway is required for maintenance of hepatic lipid homeostasis under ER stress conditions. Hepatocyte-specific knock-out of IRE1α in mice led to the development of fatty liver after treatment with an ER stress inducer through modulation of transcriptional regulators such as PPAR-γ, C/EBP-β, C/EBP-δ, and triglyceride biosynthesis-related proteins [22]. ATF6α-deficient mice fed a high-fat diet showed a tendency toward a higher degree of hepatic steatosis in association with increased expression of SREBP1c [23]. Therefore, all three UPR sensors, including PERK, IRE1α, and ATF6α, are associated with hepatic steatosis and lipid metabolism in the liver [20].

## 3. Effects of Pharmacologic Modulators on ER Stress-Induced Cellular Damage

### 3.1. Rapamycin

A well-known mTOR (mammalian target of rapamycin) inhibitor, rapamycin suppresses ER stress through activation of autophagy in various cell types. A previous study showed that ER may be a source of the membranes used for the formation of autophagic vesicles [24]. ER stress is also involved in the essential process of the autophagic pathway, as shown in the polyglutamine-induced light chain 3 (LC3) conversion [25]. ER stress-mediated autophagy may play a critical role in disposal of misfolded proteins, which cannot be cleared by ER-related degradation, thereby contributing to maintaining ER homeostasis [26,27]. Therefore, ER stress-induced autophagy may have a protective role in cell apoptosis and stress at early stages, whereas prolonged ER stress may impair autophagy, consequently leading to cell death during the development of non-alcoholic fatty liver disease (NAFLD) [28]. Zhu *et al.* suggested that rapamycin protects the liver from hepatic ischemia and reperfusion injury (IRI) by activation of autophagy through inhibition of ER stress [29]. Wang *et al.* also demonstrated that restoration of autophagy by rapamycin attenuates hepatic ER stress and insulin resistance in high fructose-fed mice [30]. Rapamycin also ameliorates adipocyte dysfunction through restoration of the palmitate-induced ER stress/NFκB-mediated pathway via stimulation of autophagy [31]. Bachar-Wikstrom *et al.* showed the presence of crosstalk between ER stress and autophagy in a rodent model of diabetes [32]. They found that treatment of diabetic Akita mice with rapamycin prevented β-cell apoptosis and improved diabetes [32]. These reports suggest that activation of autophagy by rapamycin via mTOR inhibition may be an effective pharmacological modulator of ER stress. On the other hand, Kato *et al.* found that rapamycin attenuated ER stress-induced apoptosis via selective suppression of the IRE1-JNK signaling pathway [33]. Inhibition of mTOR with rapamycin repressed ER stress and the associated apoptosis during tunicamycin treatment in renal proximal tubular cells [34]. Hwang *et al.* reported that the inhibition of mTOR by rapamycin reversed ER stress-induced insulin resistance in L6 myotubes [35].

### 3.2. Chemical Chaperones

Exogenous chemical chaperones, which mimic endogenous chaperones, reinforce the adaptive capability of the ER through the improvement of ER folding capacity, the reduction of accumulation of misfolded proteins, and promotion of mutant proteins trafficking [36]. Chemical chaperones, such as 4-phenylbutyric acid (4-PBA), trimethylamine *N*-oxide dehydrate (TMAO), and dimethyl sulfoxide (DMSO), are low-molecular-weight compounds reported to improve ER capacity and move misfolded proteins [37]. 4-PBA alleviates ER stress and prevents adipocyte differentiation through suppression of UPR activation in 3T3-L1 cells and in the adipose tissue of high fat diet-fed C57BL/6 mice [38]. Tauroursodeoxycholic acid (TUDCA), a derivative of an endogenous bile acid, has been shown to abolish thapsigargin-induced ER stress markers and subsequently alleviates apoptosis in hepatocytes [39]. Treatment with PBA and TUDCA mitigates ER stress, which is associated with improvement of insulin sensitivity in liver, muscle, and adipose tissue in *ob*/*ob* mice [40]. Furthermore, TUDCA attenuates the progression of steatohepatitis by reducing ER stress in mice fed a methionine-choline-deficient (MCD)-diet [41]. These results suggest TUDCA as a therapeutic method for ER stress-mediated liver disease [18]. 

### 3.3. AMPK Activators

AMPK-activated protein kinase (AMPK) is a primary regulator of cellular and whole body energy homeostasis. Thus, AMPK has been considered a potential therapeutic target for metabolic diseases, such as type 2 diabetes. Furthermore, it also has been reported that activation of AMPK is able to attenuate ER stress, suggesting that AMPK can be a therapeutic target for the treatment of ER stress-mediated metabolic diseases [42]. AMPK activation by 5′-aminoimidazole-4-carboxymide-1-β-d-ribofuranoside (AICAR) significantly mitigates ER stress and improves the endothelium-dependent relaxation in isolated mouse aortae [43]. In addition, AICAR contributes to protection against hypoxic injury through reduction of ER stress in cardiomyocytes [44]. Metformin, an AMPK activator, is a widely used insulin sensitizer and has been reported to protect cells against ER stress induced by palmitate. Kim *et al.* described how the protective effect of metformin may be involved in the regulation of ER stress protein expression and palmitate-induced apoptosis in HepG2 cells, thereby providing a mechanism for how metformin may ameliorate hepatic insulin resistance under hyperlipidemic conditions [45]. Furthermore, metformin also exhibits protective effects on rat insulinoma cells through the suppression of palmitate-induced phosphorylation of eukaryotic initiation factor 2 α (eIF2α), JNK, and insulin receptor substrate-1 (IRS-1) suggesting that the β-cell protective effects of metformin in lipotoxicity may be associated with the suppression of ER stress [46]. In addition, metformin attenuates renal fibrosis through suppression of ER stress via the AMPK-mediated pathway. Kim *et al.* reported that metformin suppressed tunicamycin- or thapsigargin-induced ER stress and that knockdown of AMPK with siRNA blocked the effect of metformin in tubular HK-2 cells [47]. Furthermore, metformin ameliorates high-fat diet-induced endothelial dysfunction. Cheng *et al.* demonstrated the important role of AMPK-induced PPARδ activation in suppression of ER stress and protection of endothelial function in diabetic obese mice. Therefore, they suggested metformin as a therapeutic agent for treatment of cardiovascular disease [48]. Sen *et al.* found an association between mTOR activation, ER stress, and impaired contractile function in the mammalian heart, all of which were prevented by pretreatment with rapamycin or metformin [49]. These findings support a role for metformin in the reversal of various kinds of metabolic disorders through improvement of ER stress by an AMPK-dependent mechanism. ER stress has been reported to be elevated in adipose tissue of obese humans and is known to play an important role in the integration of pathways associated with inflammation and insulin signaling in chronic metabolic diseases [50]. Alhusaini *et al.* found that lipopolysaccharide (LPS), saturated fatty acids, and hyperglycemia significantly induced ER stress, which is alleviated by salicylate, a non-steroidal anti-inflammatory drug, in human adipocytes [51]. Salicylate also activates AMPK-dynamin-related protein 1 (Drp1) to inhibit mitochondrial ROS-associated ER stress, leading to prevention of inflammation and apoptosis in the vascular endothelium [52]. Moreover, salsalate, a prodrug of salicylate, lessens carrageenan-induced insulin resistance through inhibition of selenoprotein P via AMPK-mediated suppression of ER stress in hepatocytes [53].

### 3.4. Glucagon-Like Peptide-1 (GLP-1) Receptor Agonists and Dipeptidyl Peptidase IV (DPP-IV) Inhibitors

GLP-1 is a 30-amino acid peptide hormone and an incretin derived from the transcription product of the proglucagon gene in intestinal epithelial endocrine L-cells [54]. GLP-1 appears to restore the glucose responsiveness of pancreatic β-cells and inhibit apoptosis of β-cells in freshly isolated human islets [55]. GLP-1 improves β-cell function through augmentation of mitochondrial mass and function in INS-1 rat insulinoma cells [56]. Moreover, GLP-1 also induces β-cell proliferation through transactivation of the epidermal growth factor receptor (EGFR) [57] and differentiation of pancreatic ductal cells into insulin-secreting cells [58]. The regulation of ER stress signaling by the GLP-1 pathway has also been consistently reported. Activation of the GLP-1 pathway by exenatide, a GLP-1 agonist, ameliorates ER stress via increased expression of activating transcription factor 4 (ATF4), thereby helping the cells to recover from ER stress-induced translational downregulation of insulin and improving cell survival in cultured β-cells in a PKA-dependent manner [59]. Exendin-4 protects β-cells against palmitate-induced ER stress and apoptosis through enhanced expression of cellular defense-mediated genes such as BiP, Bcl-2, and anti-apoptotic protein JunB [60]. Treatment of Akita mice, an animal model of ER stress-mediated diabetes, with exendin-4 can ameliorate ER stress-induced β-cell damage through a decrease in apoptotic cell death [61]. Prolonged high glucose and palmitate-induced ER stress and β-cell apoptosis are also attenuated by exendin-4 through regulation of SREBP1c and C/EBPβ transcription factors in INS-1 β-cells [62]. The impairment of sarco/endoplasmic reticulum Ca^2+^ ATPase (SERCA) leads to Ca^2+^ release from the ER lumen, the accumulation of misfolded proteins in the ER lumen, and consequently, the imbalance of ER homeostasis [63]. Conversely, enhanced ER SERCA activity alleviates ER stress-induced apoptosis [64,65]. Exendin-4 attenuates high glucose-induced apoptosis, which is involved in the development of diabetic cardiomyopathy, through direct suppression of oxidative stress-induced ER stress via activation of SERCA2a in neonatal rat ventricular cardiomyocytes [66]. Lee *et al.* showed that exendin-4 increases SERCA2b to maintain ER homeostasis and ameliorate palmitic acid-induced ER stress through a SIRT1-dependent pathway in hepatocytes, which may be associated with improved insulin signaling and attenuation of steatosis [67]. Moreover, exendin-4 reduced hepatocyte steatosis and improved survival by restoring ER stress response and promoting autophagy [68]. Dipeptidyl peptidase (DPP)-4 is responsible for degradation of GLP-1 [69]. DPP4 inhibitor, vildagliptin, also increased pancreatic β-cell mass and improved ER stress, possibly through C/EBPβ degradation [70]. Another DPP4 inhibitor, sitagliptin, ameliorated hepatic steatosis, inflammation, and fibrosis in mice fed a MCD diet. In this report, sitagliptin ameliorated steatohepatitis by suppression of CD36 expression, NFκB activation, lipid peroxidation, and ER stress [71]. Moreover, we observed that the novel DPP4 inhibitor, gemigliptin, protected rat cardiomyocyte H9c2 cells against ER stress-induced apoptosis and inflammation through Akt/PERK/CHOP and IRE1α/JNK-p38-mediated pathways [72]. These findings suggest that activation of the GLP-1-mediated pathway by GLP-1 receptor agonists or DPP4 inhibitors may lead to beneficial effects on cardiometabolic diseases via amelioration of ER stress.

### 3.5. Peroxisome Proliferator-Activated Receptor (PPAR) Agonists

The PPAR-α agonist fenofibrate has been reported to protect against inflammatory injury and apoptosis through alleviation of ER stress via the IRE1α-XBP1-JNK-dependent pathway in the liver of an NAFLD mouse model, which is induced by feeding a high-calorie and high-cholesterol diet (HCD) [73]. Furthermore, fenofibrate treatment also restored palmitate-induced suppression of AMPK phosphorylation and adiponectin receptor 2 (AdipoR2) expression by attenuation of ER stress and inflammation in hepatocytes [74]. Lu *et al.* reported that fenofibrate recovered endothelium-dependent vasodilatation (EDV) induced by chronic high-fat diet (HFD) feeding through decreased ER stress and increased phosphorylation of endothelial nitric oxide synthase (eNOS) [75]. PPAR-γ agonist pioglitazone protects pancreatic islets by restoring sarco-endoplasmic reticulum Ca^2+^ ATPase 2b (SERCA2b) expression, thereby suppressing ER stress and leading to improvement of β-cell function and survival [76]. Yoshiuchi *et al.* demonstrated that pioglitazone treatment reduced ER stress, which may explain the insulin sensitizing effects of pioglitazone in the liver of ER stress-activated indicator (ERAI) transgenic mice [77]. In primary human myotubes, high stearoyl-coA desaturase 1 (SCD1) inducibility was related to low ER stress and inflammatory response to palmitate [78]. On the contrary, suppression of SCD1 increases palmitate-induced ER stress and apoptosis in insulin-secreting MIN6 cells [79]. Several PPAR-γ agonists suppress apoptosis through inhibition of palmitate-induced ER stress via up-regulation of SCD1 in RAW264.7, a murine macrophage cell line [80]. PPAR-δ agonist GW501516 improves palmitate-induced ER stress, inflammation, and insulin resistance in human and mouse skeletal muscle cells through activation of AMPK and inhibition of extracellular-signal-regulated kinas 1/2 (ERK1/2) [81]. Furthermore, GW501516 has been demonstrated to ameliorate the pro-inflammatory response in human cardiac AC16 cells exposed to palmitate [82]. Palomer *et al.* reported that PPAR-δ activation by GW501516 attenuated palmitate-induced ER stress by inducing autophagy in cardiomyocytes [83]. These findings suggest that agonists of PPARs might be useful therapeutic agents to correct ER stress-mediated metabolic abnormalities.

### 3.6. Angiotensin II Type 1 Receptor Blockers (ARBs)

Angiotensin II type 1 receptor blockers (ARBs) have been widely used to control blood pressure in patients with hypertension. Wu *et al.* demonstrated that valsartan, an ARB, can ameliorate ER stress via suppression of CHOP and the p53 upregulated modulator of apoptosis (Puma)-mediated pathway in cardiomyocytes of streptozotocin-induced diabetic rats [84]. Valsartan also has been shown to have a potential nephroprotective effect on contrast media-induced renal cell apoptosis by attenuation of ER stress, which is shown as suppression of glucose-regulated protein 78 (GRP78), ATF4, and CHOP [85]. Another ARB, losartan, protects human islets from hyperglycemia-induced ER stress through inhibition of the phospholipase C-inositol 1,4,5-triphosphate-Ca^2+^ (PLC-IP3-calcium)-mediated pathway, and thereby improves β-cell function, suggesting that the renin-angiotensin system plays an important role in human β-cell physiology [86]. Sukumaran *et al.* reported that olmesartan attenuated oxidative stress, inflammatory cytokines, and ER stress in rats with experimental autoimmune myocarditis (EAM) [87]. Guan *et al.* demonstrated that enhanced ER stress might be involved in cardiomyocyte apoptosis after aortic coarctation in rats [88]. They found that telmisartan significantly attenuated ER stress, thereby reducing cardiac left ventricular hypertrophy (LVH) and improving left ventricular function [88].

## 4. Conclusions

Current literature shows that various drugs protect cells or animals from the cellular damage induced by ER stress. These drugs directly modulate not only UPR and chaperones but also mediators, including AMPK, PPARs, and Akt, that can be the therapeutic targets for treatment of ER stress-mediated diseases (Table 1). Although our understanding of the pathophysiological role of ER stress in metabolic diseases has improved in recent years, further studies are needed to address several questions. (1) To what extent are the adaptive or proapoptotic pathways of the UPR involved in the pathophysiology of ER stress-mediated diseases? [89] (2) Is systemic suppression of ER stress good for health? (3) How can we deliver the agent to the specific target tissues? (4) How can we quickly and accurately screen agents that can ameliorate ER stress? To screen effective ER stress modulators, a CHOP promoter is fused with a fluorescence gene, and a stable cell line can be constructed with this cassette. ER stress modulators may be selected by measuring suppression of the ER stress-induced fluorescence signal.

In conclusion, research to identify compounds and therapeutic strategies to control ER stress may be essential for the treatment of ER stress-associated metabolic diseases. Furthermore, extensive clinical trials are required to evaluate more effective and safer drugs to modulate ER stress in humans.

**Table 1:** Pharmacological modulators of ER stress with mediators. The arrows indicate regulation of signaling.

Category	Drug	Mediator	Effect on Disorders	Reference
mTOR inhibitors	Rapamycin	Autophagy ↑	NAFLD	[6]
			Hepatic ischemia	[7]
			Insulin resistance (hepatocyte, skeletal myocytes)	[8,13]
			Diabetes	[10]
		IRE1/JNK ↓	Apoptosis (renal cell)	[11,12]
Chemical chaperones	4-PBA	GRP78 ↓, CHOP ↓	Adipogenesis	[16]
	TUDCA	Calcium efflux ↓	Apoptosis (hepatocyte)	[17]
		eIF2α ↓, CHOP ↓	Steatohepatitis	[19]
AMPK activators	Metformin	AMPK ↑	Renal fibrosis	[26]
		AMPK ↑, PPARδ ↑	Vascular dysfunction	[27]
		eIF2α ↓, JNK ↓, IRS-1 ↓	Apoptosis (hepatocyte, endothelium)	[24,25,31]
	Salicylate/Salsalate	AMPK ↑	Apoptosis (endothelium)	[31]
			Insulin resistance (hepatocyte)	[32]
	AICAR	AMPK ↑	EDR (aortae)	[22]
			Cardiac hypoxic injury	[23]
GLP-1 receptor agonists and DPP-4 inhibitors	Exenatide	PKA ↑, ATF4 ↑, BiP ↑, Bcl2 ↑, JunB ↑, SERCA ↑, Autophagy ↑	Apoptosis (β-cell), NAFLD	[38,39,46,47]
	Vildagliptin	C/EBPβ ↓	β-cell loss	[49]
	Gemigliptin	Akt/PERK/CHOP ↓, IRE1α/JNK-p38 ↓	Apoptosis (cardiomyocyte)	[51]
PPARs agonists	Fenofibrate	IRE1α/XBP1/JNK ↓, AMPK ↑, eNOS ↑	NAFLD, EDV	[52,53,54]
	Pioglitazone	SERCA ↑, SCD1 ↑	β-cell dysfunction, Apoptosis (macrophage)	[55,59]
	GW1516	AMPK ↑, ERK1/2 ↓	Insulin resistance (skeletal myocytes)	[60]
		Autophagy ↑	Cardiac hypertrophy	[62]
ARBs	Valsartan	PUMA ↓, GRP78 ↓	Apoptosis (cardiomyocyte, renal cell)	[63,64]
	Losartan	PLC-IP3-calcium ↓	β-cell dysfunction	[65]
	Olmesartan	GRP78 ↓, CHOP ↓	Autoimmune myocarditis	[66]
	Telmisartan	GRP78 ↓, CHOP ↓	Cardiac hypertrophy	[67]

IRE1, The inositol-requiring enzyme; JNK, c-Jun N-terminal kinases; TUDCA, Tauroursodeoxycholic acid; AICAR, 5-Aminoimidazole-4-carboxamide ribonucleotide; PKA, Protein kinase A; SERCA, The Sarco/Endoplasmic Reticulum Calcium ATPase; DPP-4, Dipeptidyl peptidase-4; eNOS, endothelial nitric oxide synthase; SCD1, Stearoyl-CoA desaturase-1; ERK, Extracellular signal-regulated kinases; mTOR, Mammalian target of rapamycin; AMPK, AMP-activated protein kinase; GLP-1, Glucagon-like peptide-1; PPAR, Peroxisome proliferator-activated receptor; ARB, Angiotensin II receptor blocker; NAFLD, Non-alcoholic fatty liver disease; EDV, Endothelium-dependent vasodilation; EDR, Endothelium-dependent relaxation.
